# Child bed net use before, during, and after a bed net distribution campaign in Bo, Sierra Leone

**DOI:** 10.1186/s12936-015-0990-y

**Published:** 2015-11-18

**Authors:** Shamika Ranasinghe, Rashid Ansumana, Alfred S. Bockarie, Umaru Bangura, David Henry Jimmy, David A. Stenger, Kathryn H. Jacobsen

**Affiliations:** Department of Global and Community Health, George Mason University, 4400 University Drive 5B7, Fairfax, VA 22030 USA; Mercy Hospital Research Laboratory, Bo, Sierra Leone; Njala University, Bo, Sierra Leone; US Naval Research Laboratory, Washington, DC, 20375 USA

**Keywords:** Insecticide-treated bednets, Malaria/prevention and control, Health campaigns, Preschool children, Infants, West Africa, Sub-Saharan Africa

## Abstract

**Background:**

This analysis examined how the proportion of children less than 5-years-old who slept under a bed net the previous night changed during and after a national long-lasting insecticidal net (LLIN) distribution campaign in Sierra Leone in November–December 2010.

**Methods:**

A citywide cross-sectional study in 2010–2011 interviewed the caregivers of more than 3000 under-five children from across urban Bo, Sierra Leone. Chi squared tests were used to assess change in use rates over time, and multivariate regression models were used to examine the factors associated with bed net use.

**Results:**

Reported rates of last-night bed net use changed from 38.7 % (504/1304) in the months before the LLIN campaign to 21.8 % (78/357) during the week of the campaign to 75.3 % (1045/1387) in the months after the national campaign. The bed net use rate significantly increased (p < 0.01) from before the campaign to after the universal LLIN distribution campaign in all demographic, socioeconomic, and health behaviour groups, even though reported use during the campaign dropped significantly.

**Conclusion:**

Future malaria prevention efforts will need to promote consistent use of LLINs and address any remaining disparities in insecticide-treated bed net (ITN) use.

## Background

Malaria remains a leading cause of death among children under five years old [1]. Insecticide-treated bed nets (ITNs) have been shown to be a cost-effective intervention for reducing malaria-specific and all-cause child mortality [2–4]. A meta-analysis of four randomized controlled trials from sub-Saharan Africa found that ITN use reduced the overall under-five child mortality by 17 % in malaria-endemic areas [3]. Bed net use also reduces parasitaemia and anaemia among children [5, 6]. Despite these benefits, previous studies have shown that bed nets are not always used when young children are sleeping, even if they are available in the residence [2]. An analysis of seven national Demographic and Health Surveys (DHS) and Malaria Indicator Surveys (MIS) conducted in sub-Saharan Africa between 2001 and 2009 found that ITN ownership ranged from 27–58 %, but the last-night bed net use rates by under-five children in households that owned ITNs were only 18–56 % [6]. In a review of fifteen national DHS, MIS, and Multiple Indicator Cluster Survey (MICS) studies conducted in sub-Saharan Africa between 2003 and 2006, 4–42 % of households owned at least one ITN, but only 2–20 % of under-five children were reported to have slept under a bed net the night before the survey [7]. In households that owned ITNs, the reported last-night under-five child bed net use rates ranged from 27–71 % [7]. The results of these meta-analyses highlight the need not only to increase ownership of ITNs in malaria-endemic areas but also to increase the consistent use of bed nets by members of vulnerable populations.

The Roll Back Malaria Partnership has set a goal in malaria-endemic zones of at least 80 % of the members of high risk populations, including children less than five years old, having access to ITNs and other effective methods of malaria prevention by 2015 [8]. Over the past decade, many countries have implemented successful public health initiatives to distribute free (or reduced cost) long-lasting insecticidal nets (LLINs) to members of at-risk communities [9–13]. On 25 November 2010, a weeklong nationwide LLIN distribution campaign was launched in Sierra Leone [14]. In Bo, Sierra Leone’s second largest city, a kick-off event held in Coronation Field, the city’s main sports arena and concert venue, was followed by a week of outreach by local media and community and religious organizations. During the National Integrated Maternal and Child Health Week that ran from 26 November through 2 December, teams of trained volunteers went door-to-door in Bo and across the country distributing vouchers for free LLINs and providing children in their first five years of life with vitamin A supplements, albendazole treatment for intestinal parasites, and (at some sites) polio vaccination [13–15].

Households were eligible to receive one LLIN voucher for every two household members up to a maximum of three vouchers for households with five or more members [14]. These vouchers could be redeemed at community clinics and other distribution sites, including mobile units [13, 14]. This was a universal bed net distribution campaign [16, 17], rather than one targeted only toward specific high-risk populations such as pregnant women and young children [11]. To achieve universal coverage, and to reach the campaign’s target of at least 80 % of the population at risk for malaria sleeping under LLINs each night, more than three million LLINs needed to be distributed nationwide [14, 15]. Prior to the 2010 national LLIN campaign, the last major LLIN distribution effort in Sierra Leone had been sponsored by Médecins Sans Frontières (MSF) in 2006–2007 and involved the distribution of about 65,000 nets to vulnerable patients in the eastern part of the country, including in the city of Bo [11].

Nationally-representative surveys conducted in the years before and after the LLIN campaign showed marked improvement in bed net ownership and in bed net use by children. In the 2008 Sierra Leone Demographic and Health Survey (DHS), 26 % of children less than five years old (30 % of urban children and 24 % of rural children) were reported to have slept under a bed net the night before the survey [18]. Three years later, in a national survey of 4620 households conducted in June 2011, six months after the national LLIN distribution campaign examined in this paper, 73 % of under-fives (57 % of urban and 77 % of rural children) were reported to have slept under an LLIN the previous night [15]. The bed net use rate among bed net owners also increased, with the proportion of under-fives living in households with at least one ITN who slept under an ITN the previous night rising from 61 % in the 2008 DHS to 80 % in the 2011 post-campaign survey [15, 18]. These before-and-after comparisons provide evidence supporting the long-term success of the LLIN campaign, but these studies did not examine bed net use immediately before, during, and immediately after the bed net distribution efforts.

Bed net use surveys typically are not conducted during the launch and implementation of ITN campaigns because massive public health campaigns require the full participation of nearly all public health workers and resources. Data gathering activities during and immediately after campaign weeks focus, by necessity, on tallying the number of households visited and the number of bed nets distributed. These metrics are important for gauging coverage and, when possible, identifying under-served populations that would benefit from additional public health outreach. Household surveys are not a priority during the critical days of preparation and intense community engagement. However, data about bed net use immediately before and during campaigns may be helpful for understanding the short-term as well as the long-term effectiveness of these mass distribution efforts in increasing bed net use.

The Mercy Hospital Research Laboratory (MHRL) in Bo conducted what the lab team calls an ‘iGeode’ (integrated geography, demography, and epidemiology) study in Bo over a period of several months in 2010 and 2011. As part of a study focused on environmental health and utilization of health services, interviewers asked primary caregivers (usually mothers) in participating households a set of basic health questions about their children who were less than five years old. One of those questions about under-five children asked whether the child slept under a bed net the night before the interview. By coincidence, the national LLIN campaign for Sierra Leone was announced and implemented in the middle of the weeks of data collection in Bo. This unexpected event—a so-called ‘natural experiment’ in which events not planned by the research team may have caused changes in the behaviour of the study population [19, 20]—provided an opportunity to empirically examine how the responses to the bed net use questions changed before, during, and after the distribution of LLINs. The analysis also explores a variety of household and child characteristics that might be related to bed net use.

## Methods

### Sampling

In 2010–2011, the city of Bo, Sierra Leone, was home to 68 recognized municipal districts locally called ‘sections’. A neighbourhood is officially recognized as a ‘section’ by the municipal government once it reaches a sufficient population size and the community organizes to request formal incorporation. These 68 sections have a footprint of 30.1 km^2^ (11.6 mi^2^), and Bo has a relatively high population density for a small African city [21]. Residents within a particular section tend to share not just similar economic and occupational statuses and housing conditions, but also other characteristics such as language, tribe, and religion. After piloting the survey instrument in the two sections nearest to the MHRL research facility, a cluster random sampling method was used to select an additional 18 sections from the remaining 66 city sections across Bo. Population data from the national census bureau suggested that 20 sections would provide more than sufficient numbers of participants to have the statistical power required for the analyses of maternal, child, and environmental health that were the primary goals of the research project. A participatory geographic information system (PGIS) approach was used to create a detailed map of each of the 20 sampled sections, including identifying the location of each of the 1986 residential structures located within these 20 sections [21]. All of these residences were visited by a member of the research team, and adults from each household were invited to participate in the survey.

### Participants

Although most of the 1986 residential buildings in the sampled sections were ‘single-unit’ dwellings (not blocks of flats), the 20 sections were home to 4322 households. Adults living in the homes of their children, siblings, parents, or other relatives were often considered to be members of separate households, especially if they cooked for their own family unit separately from the other residents of the building. A consenting adult from each household within participating residences was asked to provide basic demographic and socioeconomic information about the household, and a supplemental questionnaire was used to collect basic health information about each child in the household who was younger than five years old. In total, 4306 of the 4322 (99.6 %) households in the 20 sampled sections participated in the MHRL health census. These participating households were home to 25,977 individuals. A child questionnaire was completed for 3171 of the 3196 (99.2 %) of under-five children identified as living in participating households.

### Data collection

Door-to-door interviews were conducted in the first two sections in Bo during a pilot study from 10 to 24 April 2010. Interviews were conducted in the remaining, randomly-sampled, 18 sections between 1 November 2010 and 11 February 2011. A random number generator was used to select the order in which these 18 sections were interviewed. Interviewing within each section was conducted on consecutive days until all households within the section were contacted, which in some sections meant that some data were collected in more than one of the before-, during-, and after-the-LLIN-campaign time periods. Thus, by chance, data were collected from seven sections before the LLIN distribution campaign, four sections during the campaign, and 13 sections after the campaign.

### Ethical considerations

The interviewers—MHRL staff and master’s students studying public health at a local university—all were residents of Bo and were fluent in English as well as a variety of local languages. All interviewers completed a three-day training workshop prior to beginning their fieldwork. Besides providing practical skills in interviewing techniques, data recording, and the use of handheld GPS devices, the training emphasized confidentiality, the informed consent process, respect, and other aspects of research ethics. The pilot study and the expanded study were approved by the research ethics committees of Njala University (Bo, Sierra Leone), George Mason University (Fairfax, Virginia, USA), and the U.S. Naval Research Laboratory (Washington, DC, USA). Participation was completely voluntary, and no incentives or compensation was offered to volunteers.

### Data analysis

All analyses were conducted using SPSS version 21. Of the 3171 participating under-five children, 123 (3.9 %) were missing responses for the question ‘Did this child sleep under a mosquito net last night?’ and were excluded from the analysis in this paper. Thus, the final sample size for this analysis was 3048 under-five children. Two-sided Pearson Chi squared tests with a significance level of α = 0.05 were used to examine possible differences in reported bed net use before and after the LLIN campaign within various population subgroups, such as comparing before and after rates among 2-year-olds or among those without electricity in the home.

Logistic regression models were used to examine the predictors of bed net use separately for the before-, during-, and after-the-campaign time periods. Because there were some covariates for which exposure rates differed significantly by age, such as infants (those less than 1 year old) being significantly less likely than older children to have been vaccinated for measles, dummy variables for age in years were used in all logistic regression models. Dummy variables for the surveyed sections were also included in the regression models in order to adjust for neighbourhood-level differences in socioeconomic status, population density, proximity to the centre of Bo city, and access to health resources that might not adequately be accounted for solely by household-level variables such as housing construction materials and access to utilities. For each demographic, socioeconomic, or health behaviour variable (all shown in Table 1), three different models were fit, one each for before, during, and after the LLIN campaign. Each model included the exposure of interest, age dummies, and section dummies. The *p* value for each sub-population in its own time-specific model is shown in Table 2.

**Table 1:** Factors associated with children less than 5 years old sleeping under a bed net before, during, and after the national LLIN distribution campaign in Bo, Sierra Leone

Category	Characteristic		Proportion of children with characteristic	% with characteristic reported to have slept under a bed net last night			P-value for difference in bed net use pre- to post-campaign
				Pre-campaign	Mid-campaign	Post-campaign	
Total			100.0	38.7	21.8	75.3	*<0.001*
Demographics	Child’s age (months)	0–11	20.0	42.3	26.5	74.5	*<0.001*
		12–23	17.7	38.6	18.3	76.2	*<0.001*
		24–35	20.3	37.2	25.7	75.8	*<0.001*
		36–47	21.9	40.3	14.1	76.4	*<0.001*
		48–59	20.0	34.6	24.7	73.6	*<0.001*
	Child’s sex	Female	51.1	40.2	19.9	76.0	*<0.001*
		Male	48.9	37.1	23.8	74.7	*<0.001*
Housing construction and utilities	Primary residential building material	Concrete blocks	33.6	41.9	24.0	78.6	*<0.001*
		Mud blocks or mud/sticks	66.4	37.7	19.2	73.1	*<0.001*
	Primary residential flooring material	Concrete or tile	85.6	38.3	21.8	77.2	*<0.001*
		Mud	14.4	40.9	19.2	55.6	*0.010*
	Electricity in the home	Yes	45.7	38.1	24.1	78.1	*<0.001*
		No	54.3	39.2	10.5	73.1	*<0.001*
	Approximate distance from drinking water source	<50 m	45.4	41.9	25.9	76.9	*<0.001*
		≥50 m	54.6	40.2	19.7	71.9	*<0.001*
Household health behaviour	Household treats drinking water with chemicals or filters it	Yes	64.9	46.4	23.7	73.6	*<0.001*
		No	35.1	34.2	15.8	84.5	*<0.001*
	Child reported to have had measles vaccine	Yes	88.8	38.6	22.6	78.5	*<0.001*
		No	11.2	39.0	25.0	69.4	*<0.001*
	Child’s birthplace	Healthcare facility	79.2	42.2	22.3	74.0	*<0.001*
		Home	20.8	32.0	20.5	91.7	*<0.001*

Italicized text indicates statistically significant results (p < 0.05)

**Table 2:** Odds ratios and 95 % confidence intervals for differences in bed net use among those with and without various characteristics, after adjusting for age and section (neighbourhood)

Category	Characteristic	Comparison	Pre-campaign	Mid-campaign	Post-campaign
Demographics	Child’s age (months)	12–23 vs. 0–11	1.40 (0.98, 2.01)	1.13 (0.50, 2.57)	1.07 (0.63, 1.81)
		24–35 vs. 0–11	1.30 (0.89, 1.89)	0.60 (0.24, 1.50)	1.25 (0.72, 2.18)
		36–47 vs. 0–11	1.20 (0.83, 1.74)	1.25 (0.56, 2.79)	1.64 (0.95, 2.82)
		48–59 vs. 0–11	1.36 (0.94, 1.95)	0.43 (0.18, 1.06)	1.42 (0.85, 2.39)
	Child’s sex	Male vs. female	0.92 (0.73, 1.16)	1.10 (0.63, 1.91)	1.04 (0.74, 1.47)
Housing construction and utilities	Primary residential building material	Concrete blocks vs. mud blocks or mud/sticks	1.05 (0.79, 1.39)	*1.84 (1.01*, *3.36)*	0.82 (0.57, 1.18)
	Primary residential flooring material	Concrete or tile vs. mud	0.85 (0.64, 1.13)	0.90 (0.40, 2.04)	0.67 (0.33, 1.35)
	Electricity in the home	Yes vs. no	0.94 (0.72, 1.23)	2.65 (0.87, 8.13)	*0.53 (0.36*, *0.79)*
	Approximate distance from drinking water source in meters	<50 m vs. ≥50	1.01 (0.79, 1.31)	0.93 (0.51, 1.70)	*1.51 (1.05*, *2.17)*
Household health behaviour	Household treats drinking water with chemicals or filters it	Yes vs. no	*1.71 (1.30*, *2.25)*	1.09 (0.40, 3.00)	0.64 (0.32, 1.31)
	Child reported to have had measles vaccine	Yes vs. no	1.12 (0.75, 1.67)	0.87 (0.28, 2.86)	*5.67 (2.68*, *11.99)*
	Child’s birthplace	Healthcare facility vs. home	*1.34 (1.03*, *1.72)*	1.34 (0.52, 3.46)	*0.47 (0.22*, *0.97)*

Italicized text indicates statistically significant odds ratios

## Results

Of the 3048 under-five children whose caregivers provided an answer to the question about whether the child had slept under a bed net the previous night, 1304 (42.8 %) were surveyed before the LLIN campaign, 357 (11.7 %) during the campaign, and 1387 (45.5 %) after the campaign. About 20 % of the children were in each of the five 1-year age groups (ranging from 17.7 % for 1-year-olds to 21.9 % for 3-year-olds). About half of the children were male (48.9 %) and half female (51.1 %). The participating children were from socioeconomically diverse households (Table 1). About two-thirds lived in homes built from mud blocks or mud and sticks, which are less expensive materials than the concrete blocks used for the other one-third of homes. However, only about one-sixth of the children’s homes had a mud or dirt floor rather than concrete or tile. Slightly less than half (45.7 %) of the children had electricity in their homes. Slightly more than half (54.6 %) lived in homes that were more than 50 m from the family’s drinking water source. Most (64.9 %) of the children’s families treated their water before drinking it because the water quality is poor. Most children were reported to have been vaccinated against measles (88.8 %) and to have been born at a healthcare facility (79.2 %) rather than at home.

In total, 53.4 % (1627/3048) of the under-five children whose caregivers were surveyed reported that the child had slept under a bed net the previous night. The reported bed net use rate was 38.7 % (504/1304) before the LLIN campaign, 21.8 % (78/357) during the campaign, and 75.3 % (1045/1387) after the campaign. The bed net use rate significantly decreased (p < 0.01) from before the campaign to during the campaign in all population groups. However, the last-night reported bed net use rate significantly increased (p < 0.01) from before the campaign to after the campaign in all age, sex, housing construction and utilities access, and health behaviour groups (Table 1).

Reported bed net use was similar for boys and girls in all age groups. There were no significant differences in reported bed net use by age or sex in any of the before, during, or after time periods (Table 2). Before the LLIN campaign, households reporting healthier behaviours (such as treating their drinking water and delivering babies at healthcare facilities rather than at home) were more likely than others to report that young children slept under bed nets. After the campaign, the association between bed net use and health behaviours was less clear. In regression models accounting for possible differences among the various neighbourhoods in the city, some healthy exposures (such as having had a measles vaccination) were strongly associated with bed net use but others appeared to be associated with somewhat reduced bed net use (such as being born in a healthcare facility).

## Discussion

The achievement of significantly higher rates of reported bed net use among children from all population groups indicates that the national LLIN distribution campaign in Sierra Leone in 2010 was very successful at distributing bed nets to all sectors of the Bo city population. The distribution campaign may have also reduced some inequalities in child bed net use in Bo by increasing the overall use rate and increasing the use rate in households that were previously engaging in fewer health-promoting behaviours.

These survey results point to three key conclusions. First, the LLIN campaign was very effective at increasing bed net use rates in Bo, since under-five bed net use in the MHRL citywide survey increased from less than 40 % before the LLIN campaign to 75 % after the campaign. There is also supporting evidence to suggest that these improved malaria prevention behaviours may have persisted for at least several months after the survey period ended. The MHRL survey results are similar to those collected in a national post-campaign survey in June 2011, in which 85 % of 277 under-five children from Bo (and 73 % of under-5 children nationwide) were reported to have slept under an LLIN the previous night [13, 15].

Unfortunately, two years later the 2013 Sierra Leone DHS found that only 50 % of children under five years of age nationwide had slept under a treated or untreated mosquito net the previous night (41 % of urban and 53 % of rural under-fives) [22]. This 50 % rate is considerably higher than the 26 % rate for the 2008 DHS [18], supporting the hope that the 2010 national campaign had a long-term impact on bed net use. However, the DHS findings suggest that even if the bed net use rate in Bo is higher than the national average, it is unlikely that the under-5 bed net use rate has remained at the 75 % or greater rate reported in the months immediately after the LLIN campaign. One possible contributor to a reduction in use is damage to the bed nets that may occur as a result of regular use in the months and years after a distribution campaign. A study in Gabon found that 30 % of bed nets in homes were in poor condition because of small or large holes, with 5 % of nets determined to be ‘absolutely useless’ because they had so many tears and holes that they would not provide protection from mosquito bites [23]. Members of households with damaged bed nets may decide not to sleep under them because they recognize that the nets will not confer protection against malaria. Bed net use may also decrease in the seasons when malaria incidence is lower, but the MHRL interviews were not conducted during Sierra Leone’s cold dry season [2].

Second, the LLIN campaign increased reported rates of bed net use by under-5 children from all socioeconomic strata in Bo. Although the statistical analyses in this paper suggest few disparities in bed net use in Bo before or after the LLIN distribution campaign, increasing the bed net use rate in diverse populations may have addressed any existing disparities that were not captured in the MHRL data. Most ITN campaigns find reduced disparities in post-campaign evaluations. For example, an LLIN campaign in Nigeria in 2009 found that the disparities in bed net use between wealthier and poorer quintiles disappeared after a distribution effort that increased household ownership of one or more ITNs from 10 to 70 % [12], and similar reductions in disparity by socioeconomic status were observed after a 2006 ITN distribution campaign in Kenya [10]. Given the results of this study, which are consistent with those from the June 2011 national survey in Sierra Leone, which did not find significant differences in the all-age last-night bed net use by wealth quintile [13], future malaria reduction efforts in Bo may be most effective if they make even greater efforts to reach the most disadvantaged populations.

Third, reported bed net use rates for all population groups were significantly lower during the campaign than before the campaign. There are three possible explanations for this observation: (1) the sections that were randomly assigned to be surveyed during the campaign were, by chance, neighbourhoods that happened to have very low bed net use rates compared to other sections in Bo, (2) the bed net use rate during the week of the LLIN campaign really did decrease significantly during that one week, and (3) participants surveyed during the campaign provided intentionally inaccurate answers. Since the MHRL sample size was large and it is highly unlikely that bed net use plummeted in the middle of a major health education campaign focused on the benefits of ITN use, the most likely conclusion is that participants were concerned that if they reported that they were already using bed nets they would not receive additional free nets during the distribution effort. The MHRL research team had hoped that participants would provide accurate answers to the bed net question because that survey item was embedded in a questionnaire not specifically focused on malaria and because MHRL was not directly participating in campaign outreach efforts, but this does not appear to have occurred. More generally, this result suggests that bed net use surveys during ITN campaigns (rather than before the public launch of a distribution effort) may underestimate the use rate and therefore overestimate the effectiveness of the campaign. For example, the 32 % of under-5 children who were reported to have slept under a bed net the previous night during the 2010 Multiple Indicator Cluster Survey (MICS) in Sierra Leone may be a significant underestimate, since data were collected during September through December of 2010, and that time period overlapped with the LLIN campaign [24].

These results must be interpreted cautiously because they are derived from a cross-sectional survey rather than a longitudinal or time-series study. Individual participants’ behaviours were not tracked over time. The study was observational in nature, and was not a randomized controlled trial. All responses about bed net use were reported by caregivers and not directly observed by interviewers. However, the large sample size and the application of statistical models that adjusted for differences between sections support the use of this health census dataset for examining possible differences in reported bed net use before, during, and after the nationwide bed net distribution campaign.

## Conclusions

In summary, the combined findings suggest the positive conclusion that the LLIN campaign in Bo was successful in reaching a diversity of households from across the city of Bo with free LLINs. The universal LLIN distribution campaign significantly increased bed net use immediately after the campaign. Future malaria prevention campaigns will need to address any emerging disparities in bed net use and promote continued and consistent use of LLINs in the post-campaign period. Studies of the effectiveness of mass LLIN distribution campaigns in increasing ITN use should use baseline data about bed net use rates acquired well before the launch of the distribution campaign rather than analysing data collected after the recruitment of community health volunteers or during the campaign itself, when the desire for free preventive health equipment may lead to inaccurate responses about health behaviours.

## Abbreviations

DHS: demographic and Health Survey
ITN: insecticide-treated net
LLIN: long-lasting insecticidal net
MHRL: Mercy Hospital Research Laboratory (Bo, Sierra Leone)
MIS: malaria indicator survey
MICS: multiple indicator cluster survey
MSF: Médecins Sans Frontières
PGIS: participatory geographic information system
